# Supplementary material for: The Impact of Light Wavelength and Darkness on Metabolite Profiling of Korean Ginseng: Evaluating Its Anti-Cancer Potential against MCF-7 and BV-2 Cell Lines
Source: Int J Mol Sci. 2023 Apr 24;24(9):7768. doi: 10.3390/ijms24097768 (PMC10178343; doi:10.3390/ijms24097768)
Supplement: Supplementary file 1 [file ijms-24-07768-s001.zip › ijms-2334770-supplementary.pptx]

## Slide 1
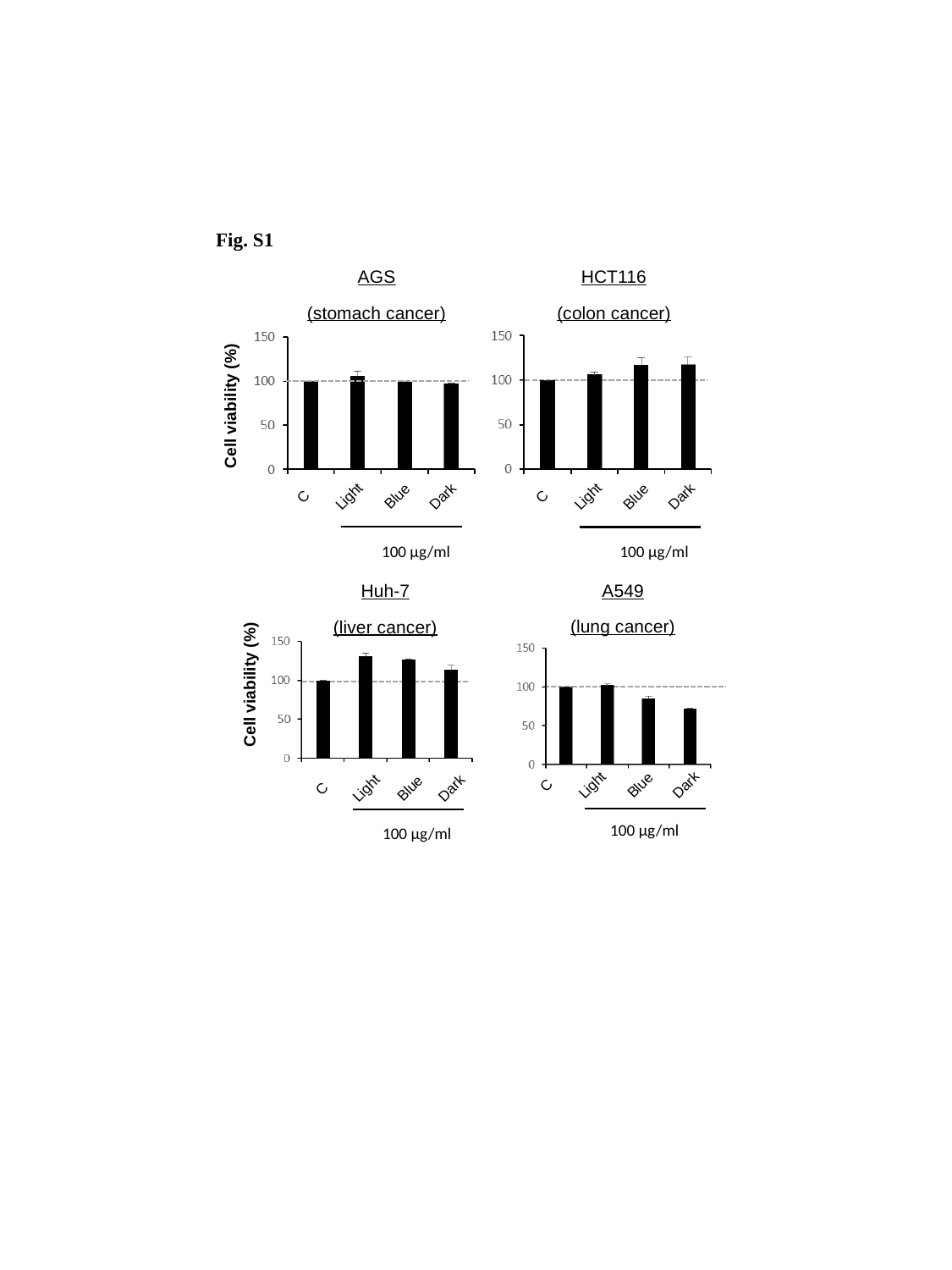

Fig. S1
AGS
(stomach cancer)
HCT116
(colon cancer)
C
Light
Blue
Dark
C
Light
Blue
Dark
 100 μg/ml
 100 μg/ml
A549
(lung cancer)
C
Light
Dark
Blue
 100 μg/ml
Cell viability (%)
Huh-7
(liver cancer)
Light
Blue
Dark
C
 100 μg/ml
Cell viability (%)

## Slide 2
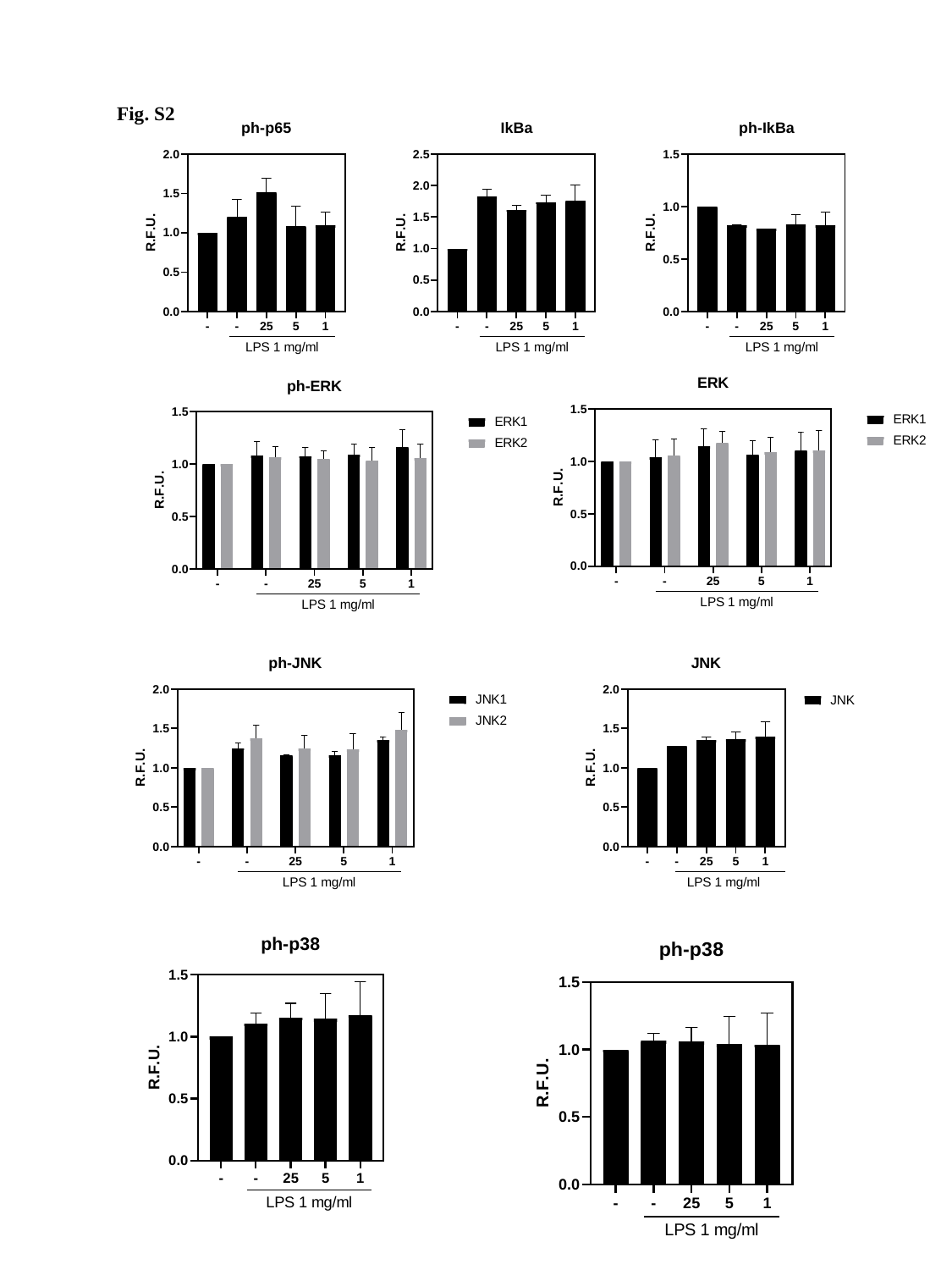

Fig. S2
